# Impact of meteorological and demographic factors on the influenza epidemic in Japan: a large observational database study

**DOI:** 10.1038/s41598-023-39617-1

**Published:** 2023-08-10

**Authors:** Genta Ito, Takahiro Takazono, Naoki Hosogaya, Naoki Iwanaga, Shogo Miyazawa, Satoki Fujita, Hideaki Watanabe, Hiroshi Mukae

**Affiliations:** 1 Data Science Department, Shionogi & Co., Ltd, Osaka, Japan; 2 Department of Respiratory Medicine, Nagasaki University Hospital, Nagasaki, Japan; 3 Biostatistics Center, Shionogi & Co., Ltd, Osaka, Japan

**Keywords:** Influenza virus, Viral infection

## Abstract

Factors affecting the start date of the influenza epidemic season and total number of infected persons per 1,000,000 population in 47 prefectures of Japan were evaluated. This retrospective observational study (September 2014–August 2019; N = 472,740–883,804) evaluated data from a Japanese health insurance claims database. Single and multiple regression analyses evaluated the time to start of the epidemic or total infected persons per 1,000,000 population with time to absolute humidity (AH) or number of days with AH (≤ 5.5, ≤ 6.0, ≤ 6.5, and ≤ 7.0), total visitors (first epidemic month or per day), and total population. For the 2014/15, 2015/16, and 2016/17 seasons, a weak-to-moderate positive correlation (R^2^: 0.042–0.417) was observed between time to start of the epidemic and time to first day with AH below the cutoff values. Except in the 2016/17 season (R^2^: 0.089), a moderate correlation was reported between time to start of the epidemic and the total population (R^2^: 0.212–0.401). For all seasons, multiple regression analysis showed negative R^2^ for time to start of the epidemic and total visitors and population density (positive for time to AH ≤ 7.0). The earlier the climate becomes suitable for virus transmission and the higher the human mobility (more visitors and higher population density), the earlier the epidemic season tends to begin.

## Introduction

Each year, the seasonal influenza virus epidemic occurs during winter in countries located at higher latitudes than in temperate regions. Influenza virus infections peak in most temperate regions around January–February in the Northern Hemisphere and around July–August in the Southern Hemisphere^1^. In general, the most unique characteristic of influenza viruses is their rapid evolution leading to their immense variability. This characteristic is attributed to the accumulation of point mutations leading to a step-by-step modification of the virus proteins, described as “antigen drift”, and the exchange of whole genome segments, described as “antigen shift” (re-assortment)^2^. In Japan, the epidemic season begins between late November and early December and ends between April and May of the following year. Apart from the recent years marked by the coronavirus disease 2019 (COVID-19) pandemic, nearly 12,000,000 people in Japan were estimated to be infected with the influenza virus in the 2018/19 season, with nearly 20,000 being hospitalized, and the excess death was approximately 3400^3^. In Japan, almost no influenza epidemics were seen in the 2020/21 and 2021/22 seasons, which is thought to be due to changes in individual behavior and the impact of public health policies^4^; however, the number of patients is increasing slightly in the 2022/23 season. Worldwide, the annual seasonal influenza epidemics result in approximately 3–5 million cases of severe illness, with 290,000–650,000 respiratory deaths^5^, leading to substantial economic impact through reduced workforce productivity and burden on healthcare services^6^.

Understanding the factors affecting influenza epidemics can help prevent and control the disease. Meteorological factors (particularly absolute humidity [AH], temperature, and amount of rainfall), different types/subtypes of influenza viruses^7–10^, and population size in temperate regions^11^ affect influenza epidemics. Additionally, the number of foreign tourists influences the epidemic mainly through droplet transmission^12,13^.

Because the diagnosis and treatment of influenza are well established and Japan has a universal healthcare system in all prefectures^14^, there is no major difference in the care-seeking behavior for influenza infections in Japan. Moreover, no previous study has simultaneously investigated the meteorological and demographic factors affecting influenza epidemics in a single-study setting. Particularly, factors influencing the time to start or magnitude of the epidemic in a single country with a universal healthcare system, by using the daily transition of influenza patients in each prefecture, have not been evaluated previously. Furthermore, understanding the characteristics of influenza epidemics is important, given the current COVID-19 and influenza pandemics.

The purpose of this first large-scale database study was to evaluate factors affecting the start date of the influenza epidemic season and the total number of infected persons per 1,000,000 population by prefecture and season, in Japan, from 2014 to 2019. The daily transition of infected persons per 1,000,000 population in each prefecture was estimated using data from a large‑scale claims database from health insurance associations, meteorological data from the Japan Meteorological Agency (JMA), demographic data from the Statistics Bureau of Japan and Japan Tourism Agency, and virus type/subtype data from the National Institute of Infectious Diseases (NIID).

## Results

### Patient population

In the JMDC database, 590,680 patients with influenza were identified and normalized to represent 8,823,761 influenza patients in the entire Japanese population in the 2014/15 season; 472,740 to 9,668,882 in the 2015/16 season; 570,423 to 9,854,278 in the 2016/17 season; 840,486 to 12,581,149 in the 2017/18 season; and 883,804 to 10,495,865 in the 2018/19 season.

In the JMDC database, from the 2016/17 season to the 2017/18 season, an approximately 47% increase in influenza cases was noted, which represented a 28% increase (after normalization) in influenza patients in Japan.

The epidemic started late in the 2015/16 season (median [minimum, maximum]: 141 [136, 150] days from September 1) and showed no major differences in the other seasons (109–116 [81, 126] days from September 1). The 10% margin for defining the epidemic season start date, that is, when the number of infected persons during the season first exceeded 10% of the maximum value, was confirmed by sensitivity analysis, presented in Supplementary Table S1 online.

The total number of infected persons per 1,000,000 population was highest in the 2017/18 season (median [minimum, maximum]), at 97,898.2 (72,274.7, 115,519.4), followed by the 2018/19 season, at 77,985.8 (55,621.2, 100,113.6), compared with that in the other seasons (range [minimum, maximum]: 65,667.9–74,381.8 [40,294.2, 103,810.8]). In 2019, the population density was approximately 6310 persons/km^2^ in Tokyo (the most populous city) and approximately 67 persons/km^2^ in Hokkaido (the least populous city). The average AH in January 2019 varied among prefectures (see Supplementary Fig. S1 online).

### Variables

The distribution of time to AH below the predefined cutoff values varied widely among the seasons (2014–2019): ≤ 5.5 (range of median [minimum, maximum]), 85–116 (44, 146) days; ≤ 6.0, 79–102 (41, 136) days; ≤ 6.5, 78–101 (39, 123) days; and ≤ 7.0, 76–90 (39, 103) days. The number of days with AH below the predefined cutoff values did not vary widely among seasons (2014–2019) at higher cutoff values (range of median [minimum, maximum] AH ≤ 6.5, 105–119 [39, 179] days and AH ≤ 7.0, 116–129 [57, 197] days) but differed substantially at lower cutoff values (AH ≤ 5.5, 65–94 [0, 163] days and AH ≤ 6.0, 89–106 [0, 165] days). The number of visitors showed an increasing trend for both the total number in the first month of the epidemic (log10: range of median [minimum, maximum]: 5.69–5.82 [5.07, 6.71]) and the average number per day during the entire epidemic period (4.21–4.30 [3.61, 5.28]; see Supplementary Table S2 online).

The total number of foreign visitors also gradually increased in every season, annually, both in the first month of the epidemic (log10: range of median [minimum, maximum]: 4.18–4.51 [3.16, 6.30]) and the average number per day during the entire epidemic season (2.70–3.03 [1.71, 4.81]; see Supplementary Table S2 online).

From 2014 to 2019, the ratio of the working-age population gradually decreased (median [minimum, maximum]) from 2.15 to 1.88 [1.47, 2.75], as did the ratio of the young population from 0.47 to 0.41 [0.27, 0.60]. The population density (persons/km^2^) remained consistent from season to season (range of median [minimum, maximum]: 2.43–2.43 [1.80, 3.29]). The epidemic viral type/subtype varied greatly by season: subtype A(H3N2) was the most prevalent subtype in most seasons (2014/15: median [minimum, maximum]: 86.87% [65.52, 100]; 2016/17: 80.52% [64.60, 100]; and 2018/19: 58.09% [38.64, 82.18]); subtype A(H1N1)pdm09 (median [minimum, maximum]: 47.25% [13.04, 71.71]), followed by type B (44.31% [20.98, 73.91]), was the most prevalent subtype in the 2015/16 season. Type B was most prevalent in the 2017/18 season (median [minimum, maximum]: 44.16% [27.27, 61.82]; see Supplementary Table S2 online).

### Correlation analysis of the time to start of the epidemic

**Figure 1:**
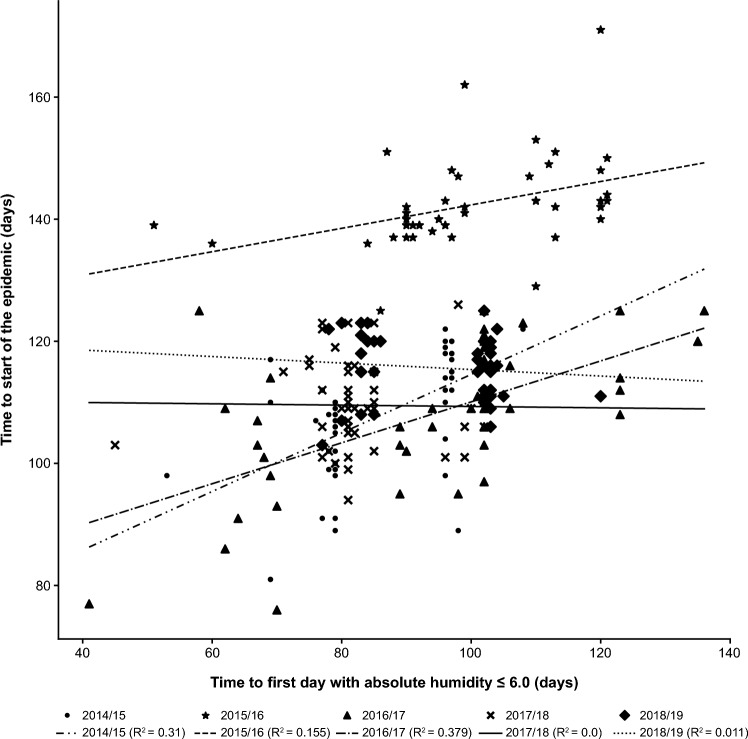
Single regression analysis showing the correlation of time to start of the epidemic with time to first day with AH ≤ 6.0. Prefectures in which the AH during the season did not fall below the cutoff value were excluded: AH ≤ 6.0, n = 46. AH, absolute humidity.

For the 2014/15, 2015/16, and 2016/17 seasons, a weak-to-moderate positive correlation (R^2^: 0.042–0.417) was observed in most of the comparisons between the time to start of the epidemic and time to AH falling below the prespecified cutoff values; no correlation was observed for the 2017/18 and 2018/19 seasons (R^2^: ≤ 0.02; Fig. 1; Table 1). The epidemic started later in the seasons when A(H1N1)pdm09 was prevalent, but no constant trend (R^2^: ≤ 0.09 for all seasons) was observed when comparisons were made between virus types/subtypes within the same season and between seasons (Table 1).

**Table 1:** Single regression analysis showing the correlation of time to start of the epidemic with time to first day with AH ≤ 5.5, ≤ 6.5, and ≤ 7.0 and proportion of virus type/subtype.

	Season	Time to first day with AH (R^2^)			Proportion of detected virus type/subtype (R^2^)		
		≤ 5.5	≤ 6.5	≤ 7.0	A(H1N1)pdm09	A(H3N2)	B
Time to start of the epidemic (days)	2014/15	0.192	0.196	0.111	0.002	0.021	0.018
	2015/16	0.168	0.169	0.080	0.021	0.022	0.053
	2016/17	0.417	0.063	0.042	0.090	0.000	0.008
	2017/18	0.014	0.001	0.001	0.023	0.026	0.001
	2018/19	0.009	0.000	0.017	0.075	0.084	0.011

Prefectures in which the AH during the season did not fall below the cutoff value were excluded: AH ≤ 5.5, n = 46; AH ≤ 6.5, n = 47 for the 2016/17 season only and n = 46 for the other seasons; AH ≤ 7.0, n = 47 for the 2016/17 and 2017/18 seasons and n = 46 for the other seasons.

AH, absolute humidity.

**Figure 2:**
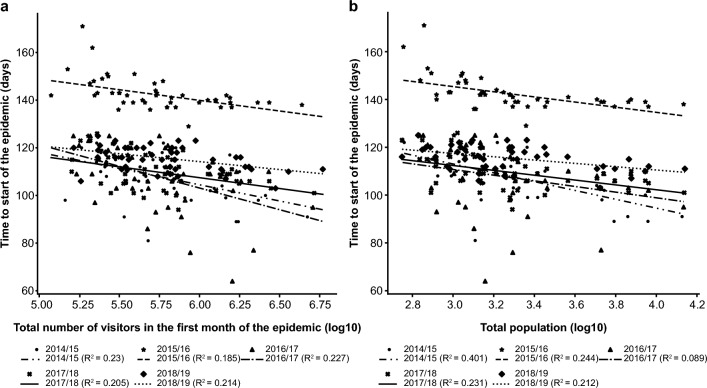
Single regression analysis showing the correlation of time to start of the epidemic with total visitors in the first month of the epidemic (**a**); and total population (**b**) (N = 47 prefectures).

For all seasons, a weak-to-moderate negative correlation was observed between the time to start of the epidemic and both the total number of visitors (R^2^: 0.185–0.230) and foreign visitors in the month of the epidemic start date (R^2^: 0.098–0.378; Fig. 2a; Table 2). Similarly, a weak-to-moderate negative correlation was observed between the time to start of the epidemic and both the ratio of the working-age population (R^2^: 0.086–0.348) and the ratio of the young population (R^2^: 0.011–0.243; Table 2). For all seasons, a weak negative correlation was observed between the time to start of the epidemic and the population density (R^2^: 0.021–0.204; Table 2). Except for the 2016/17 season (R^2^: 0.089), a moderate correlation was reported between the time to start of the epidemic and the total population (R^2^: 0.212–0.401; Fig. 2b).

**Table 2:** Single regression analysis showing the correlation of time to start of the epidemic with foreign visitors in the first month of the epidemic, ratio of working-age population, ratio of young population, and population density (N = 47 prefectures).

	Season	Number of foreign visitors in the first month of the epidemic (log10) (R^2^)	Ratio of working-age population (R^2^)	Ratio of young population (R^2^)	Population density (log10) (R^2^)
Time to start of the epidemic (days)	2014/15	0.130	0.192	0.011	0.204
	2015/16	0.098	0.086	0.013	0.068
	2016/17	0.104	0.348	0.243	0.021
	2017/18	0.164	0.164	0.123	0.152
	2018/19	0.378	0.164	0.124	0.185

In most seasons, multiple regression analysis showed no significant difference between the time to start of the epidemic and the total number of visitors in the first month of the epidemic, population density, and time to AH ≤ 7.0. Except for the 2016/17 season, the other seasons showed a negative regression coefficient for the total number of visitors in the first month of the epidemic and population density, suggesting that the time to start of the epidemic was delayed when the number of visitors and population density decreased. Similarly, in all epidemic seasons, a positive regression coefficient for time to first day with AH ≤ 7.0 suggested that the start of the epidemic was delayed when the time to AH ≤ 7.0 was extended (Table 3).

**Table 3:** Multiple regression analysis of time to start of the epidemic with total visitors in the epidemic start month, population density, and time to first day with AH ≤ 7.0 (N = 47 prefectures).

Variable	Regression coefficient	Standard error	t value	Pr >| t|
2014/15 season, adjusted R^2^: 0.400				
Intercept	124.54	27.55	4.52	< 0.0001
Total visitors (epidemic start month) (log10)	− 4.83	4.46	− 1.08	0.2853
Population density (log10)	− 10.27	3.45	− 2.97	0.0049
Time to first day with AH ≤ 7.0	0.49	0.16	3.06	0.0039
2015/16 season, adjusted R^2^: 0.221				
Intercept	173.96	18.70	9.30	< 0.0001
Total visitors (epidemic start month) (log10)	− 6.44	3.41	− 1.89	0.0659
Population density (log10)	− 3.31	2.82	− 1.17	0.2469
Time to first day with AH ≤ 7.0	0.16	0.07	2.17	0.0355
2016/17 season, adjusted R^2^: 0.224				
Intercept	213.49	32.21	6.63	< 0.0001
Total visitors (epidemic start month) (log10)	− 21.91	6.35	− 3.45	0.0013
Population density (log10)	5.42	5.08	1.07	0.2922
Time to first day with AH ≤ 7.0	0.08	0.08	0.98	0.3309
2017/18 season, adjusted R^2^: 0.181				
Intercept	153.00	17.32	8.83	< 0.0001
Total visitors (epidemic start month) (log10)	− 6.54	3.35	− 1.95	0.0577
Population density (log10)	− 3.53	2.78	− 1.27	0.2101
Time to first day with AH ≤ 7.0	0.04	0.07	0.60	0.5505
2018/19 season, adjusted R^2^: 0.195				
Intercept	143.81	14.81	9.71	< 0.0001
Total visitors (epidemic start month) (log10)	− 4.04	2.60	− 1.55	0.1277
Population density (log10)	− 3.06	2.00	− 1.53	0.1333
Time to first day with AH ≤ 7.0	0.03	0.06	0.57	0.5701

AH, absolute humidity.

### Correlation analysis of the total number of infected persons per 1,000,000 population

**Figure 3:**
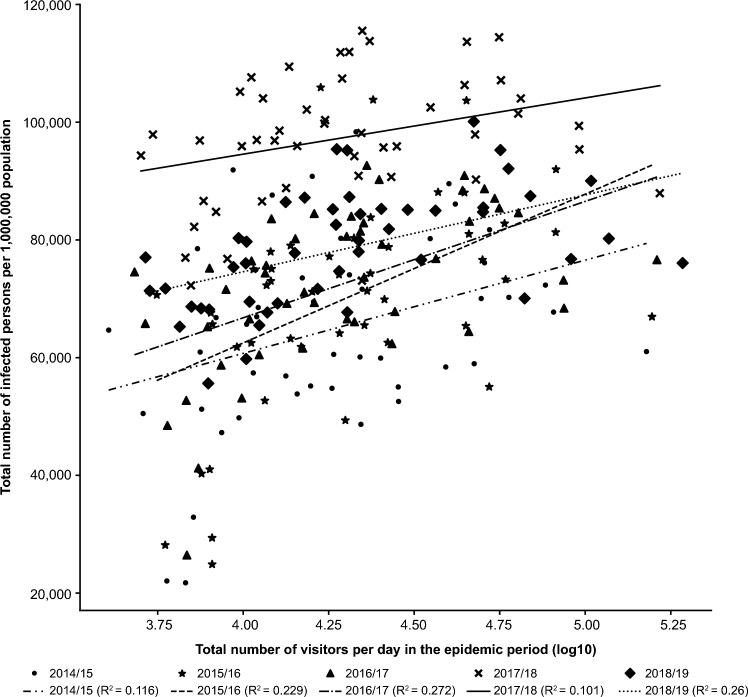
Single regression analysis showing the correlation of the total number of infected persons per 1,000,000 population with total visitors per day in the epidemic period (N = 47 prefectures).

For all seasons, a weak-to-moderate positive correlation was observed between the total number of infected persons per 1,000,000 population and both the total number of visitors (R^2^: 0.101–0.272) and foreign visitors (R^2^: 0.129–0.294) per day during the epidemic season (Fig. 3; Table 4). However, no correlation (R^2^: ≤ 0.085 for all seasons) was observed between the number of days with AH below the predefined cutoff values and the total number of infected persons per 1,000,000 population. Except in the 2017 season (R^2^: 0.069), a weak-to-moderate positive correlation was observed between population density and the total number of infected persons per 1,000,000 population (R^2^: 0.103–0.238). For all seasons, a weak-to-moderate positive correlation was observed between the total population and the total number of infected persons per 1,000,000 population (R^2^: 0.132–0.395; Table 4).

**Table 4:** Single regression analysis showing the correlation of the total number of infected persons per 1,000,000 population with number of days with AH below the cutoff, population density, total population, proportion of virus type/subtype, foreign visitors per day in the epidemic period, ratio of working-age population, and ratio of young population (N = 47 prefectures).

	Season	Number of days with AH (R^2^)				Population density (log10) (R^2^)	Total population (log10) (R^2^)
		≤ 5.5	≤ 6.0	≤ 6.5	≤ 7.0		
Total number of infected persons per 1,000,000 population	2014/15	0.049	0.039	0.030	0.028	0.117	0.173
	2015/16	0.029	0.039	0.034	0.017	0.103	0.290
	2016/17	0.004	0.013	0.011	0.008	0.238	0.307
	2017/18	0.018	0.029	0.031	0.030	0.069	0.132
	2018/19	0.085	0.070	0.044	0.036	0.157	0.395

	Season	Proportion of detected virus type/subtype (R^2^)			Foreign visitors per day in the epidemic period (log10) (R^2^)	Ratio of working-age population (R^2^)	Ratio of young population (R^2^)
		A(H1N1)pdm09	A(H3N2)	B			
Total number of infected persons per 1,000,000 population	2014/15	0.079	0.000	0.008	0.177	0.080	0.111
	2015/16	0.000	0.038	0.010	0.294	0.094	0.063
	2016/17	0.013	0.050	0.068	0.227	0.296	0.249
	2017/18	0.023	0.001	0.023	0.129	0.069	0.111
	2018/19	0.042	0.050	0.010	0.147	0.222	0.116

AH, absolute humidity.

The strength of the correlation between the total number of infected persons per 1,000,000 population and the ratios of the working-age population (R^2^: 0.069–0.296) and the young population (R^2^: 0.063–0.249) differed from season to season, but all correlations were positive (Table 4). For all seasons, no notable correlation (R^2^: ≤ 0.079) was observed between the influenza virus type/subtype and the total number of infected persons per 1,000,000 population (Table 4).

The results of multiple regression analysis showed no association between the total number of infected persons per 1,000,000 population and the total number of days with AH ≤ 7.0 (Table 5). Although significant differences in the association between the total number of infected persons per 1,000,000 population and the number of visitors per day were not noted for two seasons (2014/15 and 2017/18) during the epidemic period, the regression coefficient was positive in all seasons, indicating that prefectures with a higher number of visitors tend to have a higher total number of infected persons per 1,000,000 population. The regression coefficient with adjusted R^2^ was positive for population density for all the seasons; however, the standard error was large, and the effect of the total number of infected persons per 1,000,000 population was smaller than that of the number of visitors (Table 5).

**Table 5:** Multiple regression analysis of the total number of infected persons (per 1,000,000 population) with total visitors per day in the epidemic period, population density, and number of days with AH ≤ 7.0 (N = 47 prefectures).

Variable	Regression coefficient	Standard error	t value	Pr >| t|
2014/15: adjusted R^2^: 0.105				
Intercept	7170.23	29,114.00	0.25	0.8066
Number of visitors per day in the epidemic period (log10)	11,921.00	8008.21	1.49	0.1439
Population density (log10)	6102.25	6786.88	0.90	0.3736
Number of days with AH ≤ 7.0	− 67.30	85.17	− 0.79	0.4337
2015/16: adjusted R^2^: 0.204				
Intercept	− 43,601.00	30,873.00	− 1.41	0.1651
Number of visitors per day in the epidemic period (log10)	20,881.00	8620.32	2.42	0.0197
Population density (log10)	5705.10	6793.32	0.84	0.4057
Number of days with AH ≤ 7.0	82.06	71.15	1.15	0.2552
2016/17: adjusted R^2^: 0.282				
Intercept	− 6294.18	20,971.00	− 0.30	0.7655
Number of visitors per day in the epidemic period (log10)	14,161.00	5867.08	2.41	0.0201
Population density (log10)	8096.21	4816.35	1.68	0.1000
Number of days with AH ≤ 7.0	− 17.54	47.66	− 0.37	0.7146
2017/18: adjusted R^2^: 0.069				
Intercept	63,868.00	19,596.00	3.26	0.0022
Number of visitors per day in the epidemic period (log10)	8387.46	5279.33	1.59	0.1194
Population density (log10)	1485.05	4433.71	0.33	0.7393
Number of days with AH ≤ 7.0	− 48.93	50.47	− 0.97	0.3377
2018/19: adjusted R^2^: 0.265				
Intercept	18,253.00	14,457.00	1.26	0.2136
Number of visitors per day in the epidemic period (log10)	9807.03	3967.81	2.47	0.0175
Population density (log10)	4485.13	3433.56	1.31	0.1984
Number of days with AH ≤ 7.0	54.79	36.68	1.49	0.1425

AH, absolute humidity.

## Discussion

This is the first large-scale database study to evaluate factors affecting the start date of the influenza epidemic season and the total number of infected persons per 1,000,000 population from 2014 to 2019 in each prefecture in Japan.

An increase of approximately 28% was noted in the number of influenza cases from the 2016/17 to 2017/18 season. This increase can be attributed to the presence of multiple strains of the influenza virus (subtypes A(H1N1)pdm09 and A(H3N2), and type B in the 2017/18 season; subtypes A(H1N1)pdm09 and A(H3N2) in the 2018/19 season) as reported by the NIID and the gradual increase in the total number of visitors and foreign visitors in every season. Overall, influenza subtype A(H3N2) was the predominant subtype in most seasons, including the 2018/19 season, when a 28% increase in patients was observed after the 2016/17 season, and type B was predominant in the 2017/18 season. Single regression analysis did not show any trend related to the virus type/subtype. Notably, the population density in Japan was similar in each subsequent season, and a gradual decrease was observed in the ratio of the working-age population and the ratio of young population with each subsequent epidemic season. A negative correlation below moderate was observed between the time to start of the epidemic and ratios of both the working-age and young populations. By contrast, a positive correlation was observed between the total number of infected persons per 1,000,000 population and ratios of both the working-age and young populations. The ratios of the working-age and young populations are surrogates for the activities of daily life and intrafamilial transmission, respectively. Schools and workplaces are places where people frequently come in contact with each other. Therefore, it was anticipated that the number of infected people would increase in prefectures with higher ratios of the working-age and young populations.

Although significance was not fully demonstrated from the multiple regression analysis of the epidemic start date, in almost all seasons, the regression coefficient for the number of visitors in the epidemic start month and population density was negative, and the regression coefficient was positive for the first day with AH ≤ 7.0. The results suggest that the time to start of the epidemic was delayed when the number of visitors and population density decreased and the time to AH ≤ 7.0 increased.

However, the total number of days with AH ≤ 7.0 did not show a correlation with the total number of infected persons per 1,000,000 population in either the single or multiple regression analysis, indicating that the effect of the meteorological factors on the total number of infected persons per 1,000,000 population was limited. Particularly, the R^2^ value for most variables was small in the 2017 season, when the total number of infected persons per 1,000,000 population was high. The increase in the total number of infected persons per 1,000,000 population may be due to factors other than those investigated in this study.

Overall, these results indicate that irrespective of the season, the influenza epidemic begins earlier in prefectures with a high number of visitors and high population density, that is, high human mobility, and in prefectures with an earlier first day with AH ≤ 7.0, that is, a climate suited for influenza transmission. Given the current pandemic, population density and human flow are considered important factors for COVID-19 transmission. Influenza activity peaks earlier in years associated with the El Niño southern oscillation and/or large-scale epidemics^15^.

In almost all seasons, R^2^ values in the multiple regression analysis showed a mild-to-moderate association (R^2^: ~ 0.2–0.4). Although the time to start of the epidemic and the total number of infections can be explained, to some extent, by the factors investigated in this study, the involvement of other factors such as effect of other virus epidemics that were not assessed in this study is still likely to be substantial^1^. The R^2^ value for the multiple regression analysis was also not so large, further suggesting that the factors not investigated in this research may greatly contribute to the time to start of the epidemic and the total number of infected persons per 1,000,000 population. We also conducted a sensitivity analysis by varying the threshold values to 5%, 10%, 15%, and 20% for the epidemic season start date (when the number of infected persons during the season first exceeded 10% of the maximum value). Although changing the threshold resulted in slight variations in the regression coefficients within the same season, no substantial changes were observed that would alter the sign of the coefficients while still maintaining the significance of the regression. This finding suggests that the analytical results using a 10% threshold were robust.

Although direct comparisons cannot be made owing to differences in study design, previous studies from Japan have reported that ambient temperature and relative humidity (RH) were inversely associated with the incidence of influenza A infection, and the incidence of influenza B infection was directly associated with high RH^16^. A significant increase in the number of influenza cases was noted as AH decreased^17–19^. AH showed a strong correlation with the onset and subsidence of influenza epidemics^20^. Similarly, other reports from Japan showed that lower temperature and RH were significantly associated with higher influenza risks in more than 65% and approximately 40% of the prefectures, respectively^7,21^. A correlation between AH and isolation of influenza viruses has been demonstrated; influenza infection was prevalent at an AH of below 9 g/m^3^, and influenza prevalence showed differential occurrence by time and place.^22^ However, emerging evidence from publications on COVID-19 indicates that climate variables such as AH alone cannot account for most of the variability^23^, and it may be attributed to other factors such as viral mutations and availability of vaccines.

Previous studies using data from mainland China and Hong Kong reported a U-shaped relationship between AH and specific humidity and influenza infection rates in temperate and subtropical climates, with increased infection rates at both low and high AH/specific humidity^24,25^. The study used data from cities with latitudes ranging from 25 to 40 degrees north, and reported that there was a negative association between influenza transmissibility and AH in high latitude locations, whereas in low latitude and middle latitude locations there was a U-shaped association (higher transmissibility at both high and low AH) between transmissibility and AH^24^. Laboratory evidence shows that low AH favors influenza virus transmission and survival, supporting an explanation for influenza epidemics in winter, but it is difficult to explain the epidemic in summer when AH is high^26^. In Japan, most prefectures are located between 30 and 45 degrees north latitude and have a temperate or cool climate, whereas only Okinawa prefecture is located around 26 degrees north latitude and has a subtropical climate.

Although summer influenza epidemics have been observed in low latitude areas with high AH^27^, the fact that high AH is detrimental to influenza virus transmission and survival at the laboratory level suggests that influenza epidemics are affected by factors other than AH^26^. In the present study, which used data mainly from high-latitude regions, the influence of demographic factors that reflect human contact, such as population density and the number of visitors, on the onset of epidemics and the total number of infected persons was observed, suggesting that demographic factors are also involved in wintertime epidemics. This suggests that not only climatic factors but also demographic factors should be taken into account to explain influenza virus epidemics.

## Limitations

Our analyses were based on the number of patients that were expanded estimates from the JMDC data and may differ from the actual number of patients in Japan. Observational data derived from the prefectural capital of each prefecture were used, but the meteorological conditions may differ within the same prefecture and may largely differ from the prefectural capital in some areas. Care-seeking behavior may still be different by type of insurance program, and the proportion of patients by insurance type can also vary by prefectures. Therefore, the reliability of patient number estimates may vary from prefecture to prefecture. The variables studied in this study were those available for each prefecture at either the daily, weekly, or monthly level. Although vaccination coverage was of great interest, we could only obtain values for all of Japan at the end of the season, and data by prefecture were not available. Data on the movement of people were also of interest, but data for each prefecture were not available for the period covered by this study. These data may potentially be valuable in explaining the total number of infected persons per 1,000,000 population and the epidemic season start date. The results of this study are difficult to extrapolate to periods of strong individual behavioral change and public health policy impact from the COVID-19 pandemic but will be extrapolated eventually as the circumstances change and policies ease.

The possibility of nonlinear effects of factors, interactions, and factors other than those examined in this study was not investigated due to the limited sample size of 47 (data from each prefecture in Japan); therefore, this study is limited to an exploratory analysis of influencing factors and not evaluation of causality. In this study, we used a simple definition of the time to start of the epidemic using the number of infected persons per million population rather than the effective reproduction number, given that the estimation of the effective reproduction number can be unstable in the early stages of an epidemic. A recent study^28^ investigated the start-timing of the respiratory syncytial virus epidemic season in Japan using the effective reproduction number; a more precise analysis may be possible by investigating the relationship between the effective reproduction number or compartment model parameters such as the susceptible-exposed-infected-removed (SEIR) model^29^ and the factors used in this study.

## Conclusion

The epidemic season tended to begin earlier when human mobility was higher (more visitors and higher population density) and when the climate was conducive for virus transmission (AH ≤ 7.0). The total number of infected persons per 1,000,000 population tended to increase when human mobility was higher, but the effects of the climate suitable for virus transmission tended to be minimal. The results of this study suggest that both the start date of the epidemic and the total number of infected persons per 1,000,000 population could be explained, to some extent, by meteorological and human mobility factors investigated in this study; however, the influence of other factors that were not assessed in this study might be substantial.

## Methods

### Study design

This large, retrospective, observational database study evaluated data collected from multiple data sources between September 1, 2014, and August 31, 2019. Data on the factors likely to affect the annual influenza epidemic in 47 prefectures throughout Japan were extracted. The study was conducted in accordance with the Ethical Guidelines for Medical and Health Research Involving Human Subjects. Informed consent was not required because this study used a database containing data collected for medical claims and anonymized for secondary use. According to the guideline, ethics approval was not required because this study used publicly available statistics and a database that contained anonymized data for secondary use.

### Data sources

The estimated number of influenza cases in each prefecture and on each day of the epidemic season was estimated from the health insurance association–derived claims database provided by the JMDC (JMDC Inc., Tokyo, Japan)^30^. Throughout the study analysis period (2014/15, 2015/16, 2016/17, 2017/18, and 2018/19 seasons), the number of patients infected with influenza in each prefecture was estimated based on the claims for “influenza,” “influenza A,” or “influenza B” identified in the database; the total number of persons included in the database and the population of the entire prefecture were also recorded. These patients with influenza, influenza A, or influenza B receipt issued without suspicious flag according to Japan-specific standard diagnosis codes were defined as influenza patients and counted by the diagnosis or treatment initiation date. For each prefecture, day-to-day meteorological data, air temperature, and RH recorded at the prefectural capital were provided by the JMA^31^. Demographic and visitor data for each prefecture were obtained from the Statistics Bureau of Japan^32^ and Japan Tourism Agency^33^. Data on influenza virus types/subtypes in each prefecture and during each influenza season were provided by the NIID^34^.

The details of the method for calculating the estimated number of influenza cases by prefecture are as follows:

(1) To calculate the estimated number of patients nationwide, the population of the database was used as the denominator (A) and patients diagnosed with influenza at least once during the period were used as the numerator (B). The diagnosis rate (= B/A) was calculated by sex and age group. Thereafter, the diagnosis rate was multiplied by the Japanese population published by the government by sex and age group and considered as the estimated number of patients nationwide.

(2) To calculate the diagnosis rate (= C/D) by prefecture, by sex and age group, we used the actual number of influenza patients as the numerator (C) and all those who visited the hospital, whether diagnosed or not, as the denominator (D).

(3) Thereafter, we multiplied the diagnosis rate calculated in (2) by the population by prefecture to calculate the estimated number of patients by prefecture.

(4) The national estimate (E) was calculated by adding up the estimated number of patients by prefecture calculated in (3), and the ratio (= F/E) was calculated using that as the denominator (E) and the estimated number of patients in (1) as the numerator (F).

(5) Lastly, we multiplied the estimated value by prefecture calculated in (3) by the ratio calculated in (4), and the number calculated here is the estimated number of patients by prefecture.

### Definitions

#### Objective variable

Each year, the influenza season spans from September 1 to August 31 of the following year.

The epidemic season start date was defined as the day (after September 1) on which the number of infected persons per 1,000,000 population increased to 10% of the maximum infection in each prefecture and season, and the end date was the day on which the number of infected persons per 1,000,000 population reduced to 10% for the first time from the start date. The epidemic period is the period when the number of infected persons per 1,000,000 population exceeded 10% of the maximum infection by day in each prefecture and season.

The time to start of the epidemic (days from September 1 to the epidemic start date) in each prefecture and season was used as the first objective variable. The total number of infected persons per 1,000,000 population was the second objective variable and was evaluated for each prefecture in each season^30,32^. The shapes of the distribution of the objective variables were first checked and found to be close to a normal distribution (Supplementary Fig. S2 online). Therefore, we considered using multiple regression analysis.

### Explanatory variables

#### Meteorological factors

AH in each prefecture and season was calculated from the temperature and RH^31,35^ (see Supplementary Fig. S1 online). Temperature represents the average temperature throughout the day in the prefectural capital of each prefecture and was regarded as the average temperature for each prefecture. We first calculated the daily AH values and then processed them as a variable for each season^31^.

A previous review of global studies reported that temperature, RH, and AH are meteorological factors that affect influenza epidemics^8^. Among these factors, AH is a single indicator calculated from both temperature and RH and is considered to have a greater influence on influenza epidemics than temperature and RH^20,36^. AH of approximately 5–7 g/m^3^ favors influenza virus transmission^20^. Therefore, in this study, when the objective variable was the start date of the epidemic season, the explanatory variable was defined as the number of days from September 1 to the first day with AH below the predefined cutoff levels set (≤ 5.5, ≤ 6.0, ≤ 6.5, and ≤ 7.0). When the objective variable was the total number of infected persons per 1,000,000 population, the explanatory variable was defined as the total number of days with AH below the predefined cutoff levels set from September 1^31^.

#### Demographic factors

The total population and population density (persons/km^2^) were assessed for each prefecture as of October 1, for each season. Patients with influenza identified in the JMDC database in each season were normalized to represent the entire Japanese population. The ratios of the working-age population and young population were calculated as the ratio of the population aged 15–64 years to the population aged ≥ 65 years and that of the population aged < 15 years to the population aged ≥ 65 years, respectively, as of October 1 in each prefecture. The total number of visitors and foreign visitors on the epidemic start date (presented as log10) was the total number of visitors and foreign visitors in the first month of the epidemic. The total number of visitors and foreign visitors per day during the epidemic period (presented as log10) was calculated as the total number of visitors and foreign visitors in the epidemic period divided by the number of days in the period^32,33^.

#### Virus type/subtype

The proportion of influenza virus types (subtype A(H1N1)pdm09 and A(H3N2); and type B) was calculated from the total number of reported types/subtypes in each prefecture throughout the epidemic season^34^.

### Assessments and statistical analyses

The distribution of each objective and explanatory variable was determined for each season from 2014/15 to 2018/19. Data for each epidemic season were available for all 47 prefectures in Japan. As the epidemic start date and first day with AH less than the prespecified cutoff value varied greatly depending on the epidemic season, single and multiple regression analyses were performed for the objective and explanatory variables stratified in each season. The association (R^2^) was categorized as weak (0.1 to  ≤ 0.2), moderate (> 0.2 to  ≤ 0.5), or strong (> 0.5).

Single regression analysis was used to evaluate the correlation of time to start of the epidemic or total number of infected persons per 1,000,000 population, with time to the first day with AH ≤ 5.5, ≤ 6.0, ≤ 6.5, and ≤ 7.0 or total number of days on which the AH fell below the predefined cutoff levels stated, total number of visitors and foreign visitors (first month of the epidemic or each day in the epidemic period), ratio of the working-age population, ratio of the young population, population density, total population, and proportion of the influenza virus types/subtypes as variables.

Multiple regression analysis was used to evaluate the correlation of the time to start of the epidemic, with the total number of visitors in the first month of the epidemic, population density, and time to first day with AH ≤ 7.0 as variables. It was also used to evaluate the correlation of the total number of infected persons per 1,000,000 population, with the number of visitors per day during the epidemic period, population density, and number of days with AH ≤ 7.0 as variables. These three explanatory variables were selected for inclusion in the multiple regression analysis, equating to the recommended one-fifteenth of the sample size.

When the objective variable was the time to start of the epidemic, the first day with AH below the prespecified cutoff value during the season was used as the explanatory variable. When the objective variable was the total number of infected persons per 1,000,000 population, the total number of days with AH below the prespecified cutoff value during the season was used as the explanatory variable. Likewise, when the objective variable was the time to start of the epidemic, the total number of visitors in the first month of the epidemic was used as the explanatory variable. When the objective variable was the total number of infected persons per 1,000,000 population, the number of visitors per day during the epidemic period was used as the explanatory variable. This factor reflects the magnitude of human mobility from outside the region and within the region. Population density reflects the magnitude of human mobility within the region.

All statistical analyses were performed using SAS 9.4 (SAS Institute, Cary, NC; https://www.sas.com/, RRID:SCR_008567) and Python 3.8 (Python Software Foundation; http://www.python.org/, RRID:SCR_008394).

### Ethics approval statement

The study was conducted in accordance with Ethical Guidelines for Medical and Health Research Involving Human Subjects. According to the guidelines, ethics approval was not required because this study used publicly available statistics and a database that contained anonymized data for secondary use.

### Patient consent statement

Informed consent was not required because this study used a database that contained anonymized data collected for secondary use.

### Supplementary Information

Supplementary Information.

## Data Availability

The data that support the findings of this study are available from JMDC Inc. Restrictions apply on the availability of these data, which were used under license for this study. Data are available from the authors with the permission of JMDC Inc. For further information on obtaining the data, please contact the author, Genta Ito, at genta.ito@shionogi.co.jp.
