# Supplementary material for: Impact of meteorological and demographic factors on the influenza epidemic in Japan: a large observational database study
Source: Sci Rep. 2023 Aug 10;13:13000. doi: 10.1038/s41598-023-39617-1 (PMC10415347; doi:10.1038/s41598-023-39617-1)
Supplement: Supplementary file 1 — Supplementary Information. [file 41598_2023_39617_MOESM1_ESM.docx]

**SUPPLEMENTARY INFORMATION**

**SUPPLEMENTARY FIGURE S1** Maps showing average AH in January 2019 in all 47 prefectures in Japan with the AH calculation formula


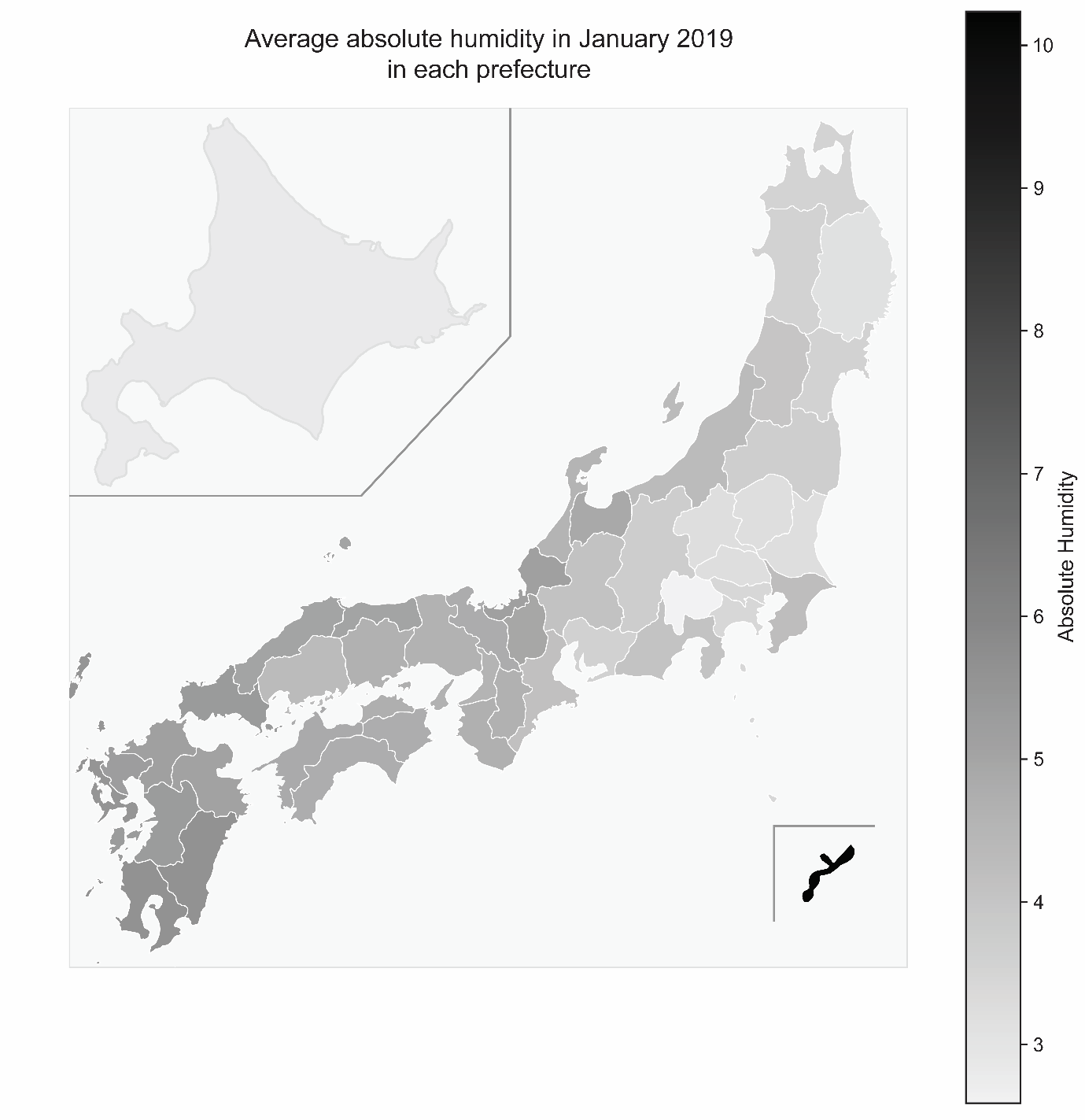


AH calculation formula^15^:

$$Saturated water vapor pressure [hPa]=6.11 *10^ (\frac{7.5*Temperature [^{\circ}C]}{237.3 + Temperature [^{\circ}C]})$$

$$Partial pressure of water vapor [hPa]=\frac{saturated water vapor pressure [hPa] * relative humidity [\%]}{100}$$

$$AH [g/m3]=\frac{217 * partial pressure of water vapor [hPa]}{(273.15 + temperature [^{\circ}C])}$$

Abbreviation: AH, absolute humidity.

Temperature represents the average temperature throughout the day in the prefectural capital of each prefecture and was regarded as the average temperature for each prefecture.

**SUPPLEMENTARY FIGURE S2** Distribution of the objective variables; epidemic season start date and total number of infected persons per 1,000,000 population.


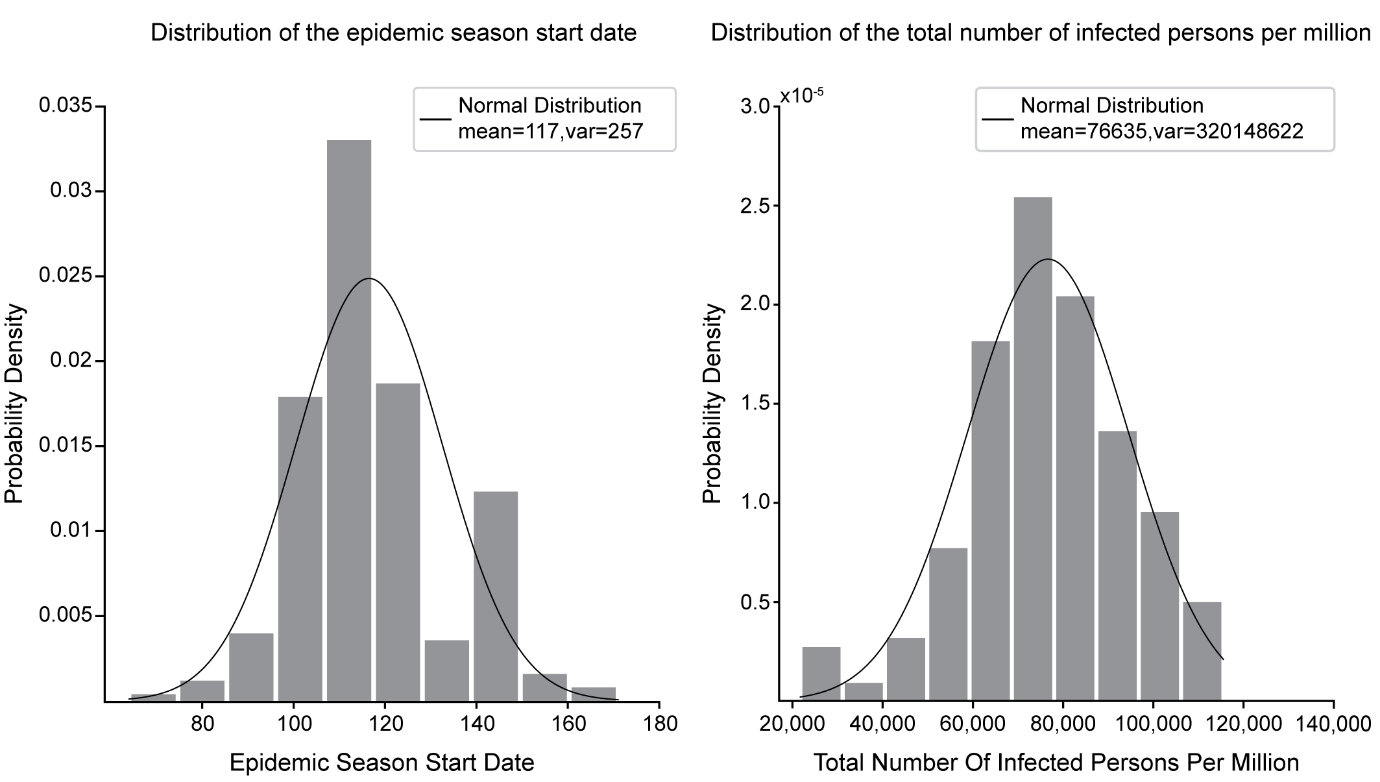


**SUPPLEMENTARY TABLE S1** Sensitivity analysis by using threshold values of 5%, 10%, 15%, and 20% to define the epidemic season start date, that is, when the number of infected persons during the season first exceeded 5%, 10%, 15%, and 20% of the maximum value

| **Season** | **Threshold of epidemic start date** | **5%** | | **10%** | | **15%** | | **20%** | |
| --- | --- | --- | --- | --- | --- | --- | --- | --- | --- |
|  |  | **Regression coefficient** | **Pr >\| t\|** | **Regression coefficient** | **Pr >\| t\|** | **Regression coefficient** | **Pr >\| t\|** | **Regression coefficient** | **Pr >\| t\|** |
| 2014/15 |  | **Adjusted R^2^: 0.487** | | **Adjusted R^2^: 0.400** | | **Adjusted R^2^: 0.371** | | **Adjusted R^2^: 0.344** | |
|  | **Intercept** | 144.3 | < 0.0001 | 124.54 | < 0.0001 | 118.68 | < 0.0001 | 111.63 | < 0.0001 |
|  | **Total visitors (epidemic start month) (log10)** | −7.77 | 0.0896 | −4.83 | 0.2853 | −3.8 | 0.3764 | −2.62 | 0.5253 |
|  | **Population density (log10)** | −11.57 | 0.0017 | −10.27 | 0.0049 | −9.19 | 0.0081 | −8.51 | 0.0109 |
|  | **Time to first day with AH ≤ 7.0** | 0.44 | 0.0078 | 0.49 | 0.0039 | 0.48 | 0.0028 | 0.49 | 0.0018 |
| 2015/16 |  | **Adjusted R^2^: 0.268** | | **Adjusted R^2^: 0.221** | | **Adjusted R^2^: 0.200** | | **Adjusted R^2^: 0.236** | |
|  | **Intercept** | 208.2 | < 0.0001 | 173.96 | < 0.0001 | 171.15 | < 0.0001 | 181.28 | < 0.0001 |
|  | **Total visitors (epidemic start month) (log10)** | −14.19 | 0.0028 | −6.44 | 0.0659 | −5.4 | 0.0984 | −6.5 | 0.067 |
|  | **Population density (log10)** | −0.49 | 0.8946 | −3.31 | 0.2469 | −3.18 | 0.2338 | −3.99 | 0.166 |
|  | **Time to first day with AH ≤ 7.0** | 0.14 | 0.1416 | 0.16 | 0.0355 | 0.14 | 0.0388 | 0.15 | 0.0394 |
| 2016/17 |  | **Adjusted R^2^: 0.369** | | **Adjusted R^2^: 0.224** | | **Adjusted R^2^: 0.327** | | **Adjusted R^2^: 0.439** | |
|  | **Intercept** | 239.27 | < 0.0001 | 213.49 | < 0.0001 | 200.29 | < 0.0001 | 150.37 | < 0.0001 |
|  | **Total visitors (epidemic start month) (log10)** | −25.98 | 0.0002 | −21.91 | 0.0013 | −19.74 | 0.0005 | −12.06 | 0.0119 |
|  | **Population density (log10)** | 0.27 | 0.9586 | 5.42 | 0.2922 | 5.29 | 0.2191 | 4.38 | 0.252 |
|  | **Time to first day with AH ≤ 7.0** | 0.13 | 0.1351 | 0.08 | 0.3309 | 0.15 | 0.0311 | 0.3 | 0 |
| 2017/18 |  | **Adjusted R^2^: 0.580** | | **Adjusted R^2^: 0.181** | | **Adjusted R^2^: 0.130** | | **Adjusted R^2^: 0.134** | |
|  | **Intercept** | 272.5 | < 0.0001 | 153 | < 0.0001 | 149.78 | < 0.0001 | 153.36 | < 0.0001 |
|  | **Total visitors (epidemic start month) (log10)** | −22.9 | < 0.0001 | −6.54 | 0.0577 | −4.74 | 0.1594 | −5.27 | 0.0881 |
|  | **Population density (log10)** | 5.5 | 0.2063 | −3.53 | 0.2101 | −3.74 | 0.1817 | −2.65 | 0.2993 |
|  | **Time to first day with AH ≤ 7.0** | −0.68 | < 0.0001 | 0.04 | 0.5505 | 0.01 | 0.9296 | 0 | 0.9876 |
| 2018/19 |  | **Adjusted R^2^: 0.154** | | **Adjusted R^2^: 0.195** | | **Adjusted R^2^: 0.158** | | **Adjusted R^2^: 0.133** | |
|  | **Intercept** | 132.13 | < 0.0001 | 143.81 | < 0.0001 | 146.84 | < 0.0001 | 143.75 | < 0.0001 |
|  | **Total visitors (epidemic start month) (log10)** | −4.21 | 0.1518 | −4.04 | 0.1277 | −5.13 | 0.058 | −5.15 | 0.0237 |
|  | **Population density (log10)** | −2.04 | 0.3603 | −3.06 | 0.1333 | −1.11 | 0.601 | 1.55 | 0.3932 |
|  | **Time to first day with AH ≤ 7.0** | 0.09 | 0.1748 | 0.03 | 0.5701 | 0.06 | 0.3491 | 0.05 | 0.3275 |

Abbreviations: AH, absolute humidity.

**SUPPLEMENTARY TABLE** **S2** Distribution of time to AH (≤ 5.5, ≤ 6.0, ≤ 6.5, and ≤ 7.0), number of visitors, foreign visitors, ratio of working-age population, ratio of young population, population density, number of days with AH (≤ 5.5, ≤ 6.0, ≤ 6.5, and ≤ 7.0), total number of visitors per day, foreign visitors per day, and virus types/subtypes

| **Median (min, max)** | **Time to AH (days)** | | | | **Total visitors in the epidemic start month (log10)** | **Foreign visitors in the epidemic start month (log10)** | **Ratio of working‑age population** | **Ratio of young population** | **Population density (persons/km^2^)**  **(log10)** |
| --- | --- | --- | --- | --- | --- | --- | --- | --- | --- |
| **N = 47** | **≤ 5.5** | **≤ 6.0** | **≤ 6.5** | **≤ 7.0** |  |  |  |  |  |
| 2014/2015 | 97  (69, 109) | 79  (53, 108) | 78  (75, 79) | 76  (75, 78) | 5.69  (5.16, 6.67) | 4.18  (3.16, 5.75) | 2.15  (1.73, 2.75) | 0.47  (0.33, 0.60) | 2.43  (1.81, 3.28) |
| 2015/2016 | 116  (84, 146) | 97  (60, 121) | 91  (85, 99) | 90  (86, 95) | 5.72  (5.07, 6.64) | 4.42  (3.39, 6.07) | 1.98  (1.58, 2.59) | 0.44  (0.30, 0.56) | 2.43  (1.81, 3.28) |
| 2016/2017 | 104  (44, 136) | 102  (41, 136) | 100  (39, 123) | 89  (39, 102) | 5.75  (5.21, 6.71) | 4.36  (3.36, 6.15) | 1.98  (1.58, 2.59) | 0.44  (0.30, 0.56) | 2.43  (1.81, 3.28) |
| 2017/2018 | 85  (79, 104) | 81  (71, 85) | 80  (71, 85) | 79  (65, 85) | 5.78  (5.20, 6.45) | 4.46  (3.46, 6.21) | 1.92  (1.47, 2.53) | 0.43  (0.27, 0.55) | 2.43  (1.80, 3.28) |
| 2018/2019 | 103  (101, 105) | 102  (77, 120) | 101  (63, 104) | 84  (60, 103) | 5.82  (5.22, 6.56) | 4.51  (3.61, 6.30) | 1.88  (1.47, 2.50) | 0.41  (0.27, 0.54) | 2.43  (1.80, 3.29) |
| **Median (min, max)** | **Number of days with AH** | | | | **Total visitors per day in the epidemic period (log10)** | **Foreign visitors per day in the epidemic period (log10)** | **Proportion of influenza virus types/subtypes** | | |
| **N = 47** | **≤ 5.5** | **≤ 6.0** | **≤ 6.5** | **≤ 7.0** |  |  | **A(H1N1)pdm09** | **A(H3N2)** | **B** |
| 2014/2015 | 94  (58, 121) | 106  (72, 136) | 115  (86, 148) | 121  (92, 160) | 4.21  (3.61, 5.18) | 2.70  (1.71, 4.57) | 0.00  (0.00, 3.59) | 86.87  (65.52, 100.00) | 11.93  (0.00, 30.00) |
| 2015/2016 | 65  (0, 137) | 89  (25, 149) | 105  (39, 168) | 116  (65, 178) | 4.28  (3.75, 4.91) | 2.90  (2.05, 4.65) | 47.25  (13.04, 71.71) | 6.44  (0.00, 15.69) | 44.31  (20.98, 73.91) |
| 2016/2017 | 94  (18, 150) | 106  (52, 154) | 112  (64, 174) | 123  (70, 197) | 4.30  (3.68, 5.21) | 2.91  (1.94, 4.70) | 2.96  (0.00, 10.94) | 80.52  (64.60, 100.00) | 13.82  (0.00, 36.36) |
| 2017/2018 | 84  (41, 136) | 95  (56, 155) | 116  (67, 159) | 127  (81, 163) | 4.28  (3.70, 4.98) | 2.99  (2.11, 4.71) | 23.08  (4.20, 38.46) | 31.93  (16.33, 46.39) | 44.16  (27.27, 61.82) |
| 2018/2019 | 66  (0, 163) | 96  (0, 165) | 119  (42, 179) | 129  (57, 193) | 4.27  (3.71, 5.28) | 3.03  (2.21, 4.81) | 35.15  (16.83, 57.95) | 58.09  (38.64, 82.18) | 6.35  (0.00, 16.36) |

Abbreviations: AH, absolute humidity; max, maximum; min, minimum.
